# Acute traumatic coagulopathy and trauma-induced coagulopathy: an overview

**DOI:** 10.1186/s40560-016-0196-6

**Published:** 2017-01-20

**Authors:** Shigeki Kushimoto, Daisuke Kudo, Yu Kawazoe

**Affiliations:** 1 Division of Emergency and Critical Care Medicine, Tohoku University Graduate School of Medicine, Seiryo-machi 2-1, Aoba-ku, Sendai, Miyagi 980-8574 Japan; 2 Department of Emergency and Critical Care Medicine, Tohoku University Hospital, Seiryo-machi 1-1, Aoba-ku, Sendai, Miyagi 980-8574 Japan

**Keywords:** Acute traumatic coagulopathy, Trauma-induced coagulopathy, Disseminated intravascular coagulation

## Abstract

Hemorrhage is the most important contributing factor of acute-phase mortality in trauma patients. Previously, traumatologists and investigators identified iatrogenic and resuscitation-associated causes of coagulopathic bleeding after traumatic injury, including hypothermia, metabolic acidosis, and dilutional coagulopathy that were recognized as primary drivers of bleeding after trauma. However, the last 10 years has seen a widespread paradigm shift in the resuscitation of critically injured patients, and there has been a dramatic evolution in our understanding of trauma-induced coagulopathy. Although there is no consensus regarding a definition or an approach to the classification and naming of trauma-associated coagulation impairment, trauma itself and/or traumatic shock-induced endogenous coagulopathy are both referred to as acute traumatic coagulopathy (ATC), and multifactorial trauma-associated coagulation impairment, including ATC and resuscitation-associated coagulopathy is recognized as trauma-induced coagulopathy. Understanding the pathophysiology of trauma-induced coagulopathy is vitally important, especially with respect to the critical issue of establishing therapeutic strategies for the management of patients with severe trauma.

## Background

Trauma remains a leading cause of death and permanent disability in adults despite advances in systematic approaches including prevention, resuscitation, surgical management, and critical care [1]. Trauma-related death and disability have also been suggested to have a great impact on global productivity.

Bleeding accounts for 30–40% of all trauma-related deaths and typically occurs within hours after injury [2]. Although the mortality of trauma patients requiring massive transfusion exceeds 50% [3], at least 10% of deaths after traumatic injury are potentially preventable, and 15% of those are due to hemorrhage; many of these deaths occur within the first few hours of definitive care, with coagulopathy playing a crucial role [4–6].

Regarding the management of patients requiring massive transfusion, it has been repeatedly suggested that patients are more likely to die from intraoperative metabolic failure than from the failure to complete organ repairs [7, 8]. Coagulopathy is one of the most preventable causes of death in trauma and has been implicated as the cause of almost half of hemorrhagic deaths in trauma patients [8, 9].

Previous landmark studies identified iatrogenic and resuscitation-associated causes of coagulopathic bleeding after traumatic injury, of which hypothermia, metabolic acidosis, and dilutional coagulopathy were recognized as primary drivers of bleeding after trauma [9–11]. However, endogenous acute coagulopathy, which occurs within minutes following injury, before and independent of iatrogenic factors, is clearly recognized and accepted as the primary cause of perturbed coagulation after injury [12]. Coagulopathy is present at the time of admission to the emergency department in up to 25–35% of trauma patients [9, 10, 13]. Understanding the pathophysiology of trauma-induced coagulopathy is vitally important, especially with respect to the critical issue of establishing therapeutic strategies for the management of patients with severe trauma [14].

### Coagulopathy in the acute phase of trauma: not a simple dilutional and resuscitation-related coagulopathy

Coagulopathy in the acute phase of trauma has long been known to coexist with severe hemorrhage and has been recognized as a co-phenomenon and unavoidable sequela of resuscitation for patients requiring massive transfusion, and accompanied by hypothermia, metabolic acidosis, and dilutional coagulopathy. However, our understanding of the mechanisms and clinical importance of coagulopathy changed significantly after the identification of an endogenous coagulation abnormality, i.e., acute traumatic coagulopathy (ATC), nearly a decade ago [9, 10]. The presence of this impairment early after trauma has been demonstrated to be an independent predictor for increased organ dysfunction, infection, and overall mortality [15]. Trauma itself and/or traumatic shock can directly induce endogenous ATC, in contrast with the indirect mechanisms such as hypothermia, metabolic acidosis, and dilutional coagulopathy [16–18]. These contributing factors of hemostatic impairment exacerbate ATC and may participate collectively to the clinical features of trauma-induced coagulopathy [16–18]. Acute coagulopathy has recently been identified at admission before trauma resuscitation in one in four trauma patients [10, 13, 19], and is associated with a fourfold increase in mortality [9, 10, 13, 19].

**Fig. 1:**
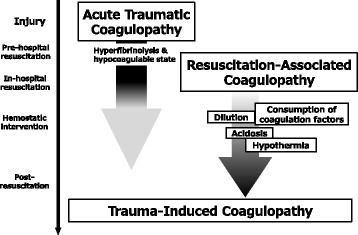
Time phase of two components of trauma-induced coagulopathy following injury: acute traumatic coagulopathy (ATC) and resuscitation-associated coagulopathy. Endogenous ATC caused by trauma itself and traumatic shock presents immediately after injury and continue during resuscitation phase. Resuscitation-associated coagulopathy, involving hypothermia, metabolic acidosis, and dilutional coagulopathy, aggravates the ATC accompanied with therapeutic resuscitation and continue to post-resuscitation phase

Coagulopathy in the acute phase of trauma patients consists of two core components: (1) trauma itself and/or traumatic shock-induced endogenous ATC and (2) resuscitation-associated coagulopathy [20] (Fig. 1).

Although no consensus has been reached regarding a definition and there are different approaches to the classification and naming of trauma-associated coagulation impairment, in this manuscript, we define ATC as trauma itself (directly trauma-induced) and/or traumatic shock-induced endogenous ATC and trauma-induced coagulopathy as multifactorial trauma-associated coagulation impairment, including ATC and resuscitation-associated coagulopathy associated with hypothermia, metabolic acidosis, and dilutional coagulopathy [11, 18]. Gando and Hayakawa summarized the important components of trauma-induced coagulopathy, consisting of endogenously (trauma- and traumatic shock-induced) primary pathologies and exogenous secondary pathologies (Table 1) [21].

**Table 1:** Summary of trauma-induced coagulopathy (cited from [21])

1 Physiological changes • Hemostasis and wound healing 2 Pathological changes • Endogenously induced primary pathologies o Disseminated intravascular coagulation (DIC) • Activation of coagulation • Insufficient anticoagulation mechanisms • Increased fibrin(ogen)olysis (early phase) • Suppression of fibrinolysis (late phase) • Consumption coagulopathy o Acute coagulopathy trauma-shock (ACOTS) • Activated protein C-mediated suppression of coagulation • Activated protein C-mediated increased fibrinolysis • Exogenously induced secondary pathologies that modify DIC and ACOTS o Anemia-induced coagulopathy o Hypothermia-induced coagulopathy o Acidosis-induced coagulopathy o Dilutional coagulopathy o Others	

Cap and Hunt classified trauma-associated coagulopathies into three phases [11]. The first phase is immediate activation of multiple hemostatic pathways, with increased fibrinolysis, in association with tissue injury and/or tissue hypoperfusion. The second phase involves therapy-related factors during resuscitation. The third, post-resuscitation, phase is an acute-phase response leading to a prothrombotic state predisposing to venous thromboembolism.

**Fig. 2:**
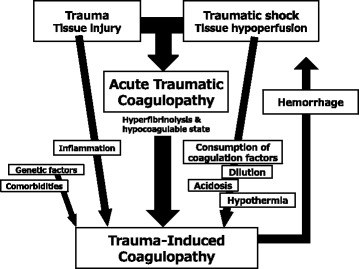
Trauma-induced coagulopathy and acute traumatic coagulopathy (ATC). Trauma itself and/or traumatic shock-induced endogenous ATC are referred to as ATC, and multifactorial trauma-associated coagulation impairment, including ATC and resuscitation-associated coagulopathy involving hypothermia, metabolic acidosis, and dilutional coagulopathy, is termed trauma-induced coagulopathy

Of these three phases, the first phase corresponds to ATC, and the clinical features of the first phase along with the pathophysiologic factors of the second phase provide the characteristics of trauma-induced coagulopathy (Fig. 2) [22]. Recently, the clinical features and pathophysiology of trauma-induced coagulopathy have been recognized as the comprehensive condition of ATC involving resuscitation-associated coagulopathy, a systemic inflammatory response to tissue injury, and predisposing factors [23]. Currently recommended management lists for the first and second phases based on The European guideline on management of major bleeding and coagulopathy are summarized as Table 2 [24]. It is also recommended that early mechanical thromboprophylaxis with intermittent pneumatic compression or anti-embolic stockings followed by pharmacological thromboprophylaxis within 24 h after bleeding has been controlled [24].

**Table 2:** Currently recommended management for trauma-induced coagulopathy (cited from [24] with modification)

Initial assessment and management
Extent of traumatic hemorrhage assessed
Patient in shock with identified source of bleeding treated immediately
Patient in shock with unidentified source of bleeding sent for further investigation
Coagulation, hematocrit, serum lactate, base deficit assessed
Antifibrinolytic therapy (tranexamic acid within 3 h after injury) initiated
Patient history of anticoagulant therapy assessed
Resuscitation
Systolic blood pressure of 80–90 mmHg achieved in absence of traumatic brain injury
Measures to achieve normothermia implemented
Target hemoglobin level 7–9 g/dl achieved
Surgical intervention
Damage control surgery performed in hemodynamically unstable patient
Coagulation management
Massive transfusion protocol with high plasma: red blood cell ratio employed
Target fibrinogen level 1.5–2 g/l achieved
Target platelet level achieved
Prothrombin complex concentrate administered if indicated due to vitamin K antagonist, oral anticoagulant or evidence from viscoelastic monitoring

### Pathophysiology of ATC and its clinical impact on patients with severe trauma

Although the pathophysiology of coagulation impairment in the acute phase of trauma has not yet been elucidated, ATC plays a pivotal role. It has been repeatedly demonstrated that ATC is a frequent complication in patients with severe trauma [9, 10, 13, 25].

### Clinical features of ATC

ATC that is caused by trauma-induced tissue injury and/or traumatic shock (generalized tissue hypoperfusion) presents as systemic activation of coagulation responses associated with increased fibrinolysis [19, 26, 27]. The clinical features of ATC can be summarized as follows [11, 18, 28–30]:

- Increased activation of coagulation (as a background pathophysiologic characteristic) leading to uncontrolled coagulation
- Coagulation impairment secondary to coagulation factor deficiency (consumption coagulopathy) leading to a hypocoagulable state
- Increased fibrin(ogen)olysis

The increased fibrin(ogen)olysis constitutes the most prominent feature of ATC.

### Clinical impact of ATC

Coagulopathy in trauma patients is associated with higher transfusion requirements, longer intensive care unit and hospital stays, prolonged mechanical ventilation support, and a greater incidence of multiple organ dysfunction. Compared with patients without coagulopathy, those with coagulopathy have a three- to fourfold greater mortality and up to eight times higher mortality within the initial 24 h of injury [9, 10, 31, 32].

### Mechanisms of ATC

It has been argued that activated protein C plays a central role in the mechanism of ATC. In initial observations in trauma patients with systemic hypoperfusion, defined by an elevated base deficit, a correlation was found between ATC and increased levels of activated protein C, reduced levels of protein C, and elevated soluble thrombomodulin [31]. The activation of the thrombomodulin-protein C system has been suggested as a principle pathway mediating ATC, characterized as hyperfibrinolysis and a hypocoagulable state, and this proposed mechanism is distinct from clotting factor consumption or dysfunction [31, 33].

However, authors only speculated increase in the levels of activated protein C based on the lower levels of protein C. The precise pathophysiologic mechanisms are still under investigation. Other mechanisms have been suggested and may contribute to this pathologic condition [34, 35].

### ATC is mediated by dysregulated activation of the thrombomodulin-protein C system

#### Physiologic response to tissue injury by the thrombomodulin-protein C system

In physiologic conditions, tissue injury leads to thrombin generation and fibrin and clot formation through the extrinsic coagulation pathway. Although the clotting process is initially localized at the site of injury, systemic activation of coagulation secondary to the escape of thrombin from the injury site is inhibited by circulating antithrombin or by the binding of thrombin to constitutively expressed thrombomodulin on intact endothelial cells [36]. Protein C is converted from an inactive to an active form by the complex of thrombin with thrombomodulin on the endothelial cell surface. Activated protein C serves a protective function to maintain tissue perfusion by inhibiting thrombosis through inactivation of factors Va and VIIIa and inhibiting plasminogen activator inhibitor-1 (PAI-1) during periods of decreased flow [33, 37].

#### Tissue hypoperfusion due to traumatic shock and activation of protein C

Sustained tissue hypoperfusion is associated with elevated levels of soluble thrombomodulin secondary to endothelial damage, which can increase the availability of thrombomodulin to bound thrombin [31]. As a result of complex formation with thrombomodulin, the role of thrombin can be diverted from procoagulant to anticoagulant by excess activation of protein C [31, 38]. This hypothetical condition has been named acute coagulopathy of trauma-shock (ACOTS) [39, 40]. Although the precise pathophysiology remains to be elucidated, these mechanisms may lead to the hyperfibrinolytic state in patients with ATC, which is reflected in increased tissue plasminogen activator (t-PA), decreased PAI, and increased d-dimer levels [31, 33].

### ATC as disseminated intravascular coagulation with a fibrinolytic phenotype

Disseminated intravascular coagulation (DIC) is characterized by activation of the tissue factor-dependent coagulation pathway and insufficient anticoagulant mechanisms, leading to consumption of platelets and coagulation factors and associated with coagulopathic clinical features [41–43]. The Scientific and Standardization Committee (SSC) on DIC of the International Society on Thrombosis and Haemostasis (ISTH) defined DIC as follows: DIC is an acquired syndrome characterized by the intravascular activation of coagulation with loss of localization arising from different causes. It can originate from and cause damage to the microvasculature, which, if sufficiently severe, can produce organ dysfunction. The most important points of the definition of DIC are “intravascular activation of coagulation with loss of localization” and “damage to the microvasculature”, which means thrombin generation and its activation in the circulation and extensive damage to the microvascular endothelium give rise to insufficient coagulation control [18, 44].

Although there is no consensus regarding the classification of the pathophysiology and clinical features of DIC, it may be divided into fibrinolytic and antifibrinolytic phenotypes [41–43]. The characteristics of ATC are essentially the same as DIC with the fibrinolytic phenotype, which contributes to massive bleeding and patients’ prognoses [45, 46]. DIC in the late phase of trauma is a thrombotic phenotype, which can be complicated with the development of multiple organ dysfunction syndrome [43, 47, 48].

The synergistic activation of primary and secondary fibrin(ogen)olysis causes DIC with the fibrinolytic phenotype [42, 46], whereas both depression of the inhibitory system of coagulation and PAI-1-mediated inhibition of fibrinolysis cause DIC with the thrombotic phenotype [41, 42].

The Scientific and Standardization Committee on DIC of ISTH commented on two concepts regarding the hemostatic changes occurring early after trauma: DIC with the fibrinolytic phenotype and coagulopathy of trauma (COT) and ACOTS. Although there are differences between these two conditions and more information is needed to elucidate the pathogenesis of these entities, it has been suggested that COT/ACOTS is not a new concept but a disease entity similar to or the same as DIC with the fibrinolytic phenotype [49].

### Acute traumatic coagulopathy may not be a DIC

DIC is defined as a clinicopathologic syndrome characterized by widespread activation of coagulation resulting in intravascular formation of fibrin and thrombotic occlusion of vessels [50, 51]. Almost all severely traumatized patients, especially those with ATC, are diagnosed as having DIC according to the scoring systems of the ISTH and Japanese Association for Acute Medicine [48, 52, 53]. However, no anatomopathologic evidence, e.g., intravascular formation of fibrin and thrombotic occlusion of vessels, has been demonstrated, and consumption coagulopathy leading to platelet and coagulation factor deficiency is not a common finding in patients with ATC [27].

Rizoli and colleagues reported the relationship between a clinical diagnosis of DIC using the ISTH score and pathologic findings in a prospective observational cohort study of severely injured patients (injury severity score ≥16) [53]. All organs surgically removed within 24 h of trauma were reviewed by two independent pathologists. All autopsy reports were also reviewed. Because d-dimer levels have a disproportional influence in trauma DIC scores, most patients have DIC scores that indicate overt DIC or are suggestive of DIC within 24 h of trauma. However, decreased platelet counts, fibrinogen levels, clotting times, and factor VIII levels were not evident. In addition, no anatomopathologic evidence of DIC was identified in the first 24 h, even after additional histochemical staining, in 40 excised organs and 27 autopsy reports.

Although diffuse intravascular fibrin formation and deficiencies in coagulation factors are suggested to be specific findings for DIC, these clinical and pathologic features were not observed in patients with ATC. Therefore, the pathophysiologic mechanism of ATC has been emphasized as being different from that of DIC [13, 27, 41, 54]. However, thrombin generation with marked decrease in fibrinogen and d-dimer elevation was observed [13, 27, 41, 54], suggested to be consistent with the pathophysiology of DIC.

DIC with the fibrinolytic phenotype as a pathophysiologic mechanism for ATC has been definitively denied by researchers emphasizing activation of the thrombomodulin-protein C system as a principle pathway mediating ATC [43]. Some researchers suggested that ATC is not a DIC because there is no clear evidence of diffuse anatomopathologic intravascular fibrin deposition and also because the “DIC hypothesis with a fibrinolytic phenotype” is a confusion of terms and should be abandoned. They suggested that a state in which fibrinolytic activity exceeds the capacity of the hemostatic system to make stable clots, resulting in excess or uncontrolled hemorrhage, should be termed systemic activation of fibrinolysis with poor hemostasis [27]. However, they misunderstand the concept of DIC, leading to inappropriate conclusion. DIC is intravascular activation of coagulation with loss of localization and damage to the microvasculature, which means thrombin generation, not fibrin clot formation and its activation in the circulation and extensive damage to the microvascular endothelium that give rise to insufficient coagulation control [18, 44].

Trauma-induced coagulopathy, especially ATC, is a dynamic entity that evolves over time, and it has been suggested that no single hypothesis explains the different manifestations of coagulopathy [27]. Many problematic issues have been suggested regarding the activation of the thrombomodulin-protein C system mechanism, and a pathophysiologic overlap with DIC has also been proposed in recent reviews [18, 55].

### Pathophysiologic mechanism of increased fibrinolysis in ATC

ATC presents as systemic activation of coagulation associated with increased fibrinolysis [19, 26, 27], and the increased fibrin(ogen)olysis is the most characteristic feature.

Thrombin is a central molecule in hemostasis. Thrombin generation converts fibrinogen to fibrin, resulting in fibrin strand formation, and activates platelets, leukocytes, and endothelium. However, thrombin also stimulates the production of t-PA from the endothelium, an effect previously known as secondary fibrinolysis. Stimulation of t-PA release from the endothelium by other factors such as hypoxia, adrenaline, and vasopressin is known as primary fibrinolysis [11]. Traumatic shock-induced tissue hypoperfusion has also been demonstrated to promote the production of t-PA from the endothelium, and increased t-PA levels have been reported in coagulopathic trauma patients [42, 56].

Additionally, it has been demonstrated that fibrin(ogen)olysis is accelerated by α2-plasmin inhibitor deficiency secondary to increased plasmin production [30]. These multiple factors are suggested to contribute to the fibrinolytic status in patients with severe trauma.

The critical point in the pathogenesis of fibrinolysis in patients with ATC is the difference in timing of onset between the immediate t-PA release from the endothelium and later expression of PAI-1 mRNA, which results in an extreme imbalance of these molecules [43, 57, 58]. The difference of several hours may play an important role in the fibrinolytic condition. This difference in timing is supported by the findings that the levels of PAI-1 are identical immediately after trauma in almost all severely traumatized patients regardless the diagnosis of DIC, whereas the levels of t-PA and plasmin generation were both significantly increased in patients diagnosed as having DIC [41, 59–61].

## Conclusions

Exsanguinating hemorrhage is the most common preventable cause of death after trauma [7, 62, 63]. Many of these deaths occur within the first few hours of definitive care, with coagulopathy playing a major role. A widespread paradigm shift in the resuscitation of critically injured patients with hemorrhagic shock has changed the management of severe trauma from a definitive surgical approach to damage control surgery during the past two decades [7, 62, 63]. Rewarming efforts, early correction of acidosis, and aggressive crystalloid resuscitation in patients requiring damage control surgery have been the prime tenets of a trauma resuscitation strategy. This focus on early correction of physiologic abnormalities has prompted the era of damage control surgery [17, 20, 23, 64–68]. However, improvement of clinical outcomes in patients requiring damage control surgery, even accompanied by aggressive correction of physiologic derangements, is still insufficient.

Although trauma-induced coagulopathy, consisting of ATC and resuscitation-associated coagulopathy, is multifactorial, it is definitively the most important issue for the management of severe trauma patients. Damage control surgery accompanied by sophisticated damage control resuscitation [17, 69, 70], including hypotensive/hypovolemic resuscitation and hemostatic resuscitation based on an understanding of the pathophysiology of ATC and trauma-induced coagulopathy, must be the central theme of the management of severely traumatized patients with ATC.

## Abbreviations

ACOTS: Acute coagulopathy of trauma-shock
ATC: Acute traumatic coagulopathy
COT: Coagulopathy of trauma
DIC: Disseminated intravascular coagulation
ISTH: International Society of Thrombosis and Haemostasis
PAI: Plasminogen activator inhibitor
t-PA: Tissue plasminogen activator
